# Identification of long non-coding transcripts with feature selection: a comparative study

**DOI:** 10.1186/s12859-017-1594-z

**Published:** 2017-03-23

**Authors:** Giovanna M. M. Ventola, Teresa M. R. Noviello, Salvatore D’Aniello, Antonietta Spagnuolo, Michele Ceccarelli, Luigi Cerulo

**Affiliations:** 1 Department of Science and Technology, University of Sannio, via Port’Arsa, 11, Benevento, 82100 Italy; 2 BioGeM, Institute of Genetic Research “Gaetano Salvatore”, c.da Camporeale, Ariano Irpino (AV), 83031 Italy; 3 Biology and Evolution of Marine Organisms, Stazione Zoologica Anton Dohrn, Villa Comunale, Napoli, 80121 Italy

**Keywords:** lncRNA, Feature selection, Classification

## Abstract

**Background:**

The unveiling of long non-coding RNAs as important gene regulators in many biological contexts has increased the demand for efficient and robust computational methods to identify novel long non-coding RNAs from transcripts assembled with high throughput RNA-seq data. Several classes of sequence-based features have been proposed to distinguish between coding and non-coding transcripts. Among them, open reading frame, conservation scores, nucleotide arrangements, and RNA secondary structure have been used with success in literature to recognize intergenic long non-coding RNAs, a particular subclass of non-coding RNAs.

**Results:**

In this paper we perform a systematic assessment of a wide collection of features extracted from sequence data. We use most of the features proposed in the literature, and we include, as a novel set of features, the occurrence of repeats contained in transposable elements. The aim is to detect signatures (groups of features) able to distinguish long non-coding transcripts from other classes, both protein-coding and non-coding. We evaluate different feature selection algorithms, test for signature stability, and evaluate the prediction ability of a signature with a machine learning algorithm. The study reveals different signatures in human, mouse, and zebrafish, highlighting that some features are shared among species, while others tend to be species-specific. Compared to coding potential tools and similar supervised approaches, including novel signatures, such as those identified here, in a machine learning algorithm improves the prediction performance, in terms of area under precision and recall curve, by 1 to 24%, depending on the species and on the signature.

**Conclusions:**

Understanding which features are best suited for the prediction of long non-coding RNAs allows for the development of more effective automatic annotation pipelines especially relevant for poorly annotated genomes, such as zebrafish. We provide a web tool that recognizes novel long non-coding RNAs with the obtained signatures from fasta and gtf formats. The tool is available at the following url: http://www.bioinformatics-sannio.org/software/.

**Electronic supplementary material:**

The online version of this article (doi:10.1186/s12859-017-1594-z) contains supplementary material, which is available to authorized users.

## Background

The recent advances in whole transcriptome sequencing offers new opportunities for discovering novel functional transcript elements. In past decades only 2% of mammalian genome have been identified as coding for proteins, while it is now known that a significant amount of the genome can be transcribed into different families of non-coding RNAs (ncRNAs) [1]. Such a high amount of transcripts demanded for the development of methods able to detect functional ncRNAs, and, among them, long non-coding RNAs (lncRNAs) which have emerged as important regulators of gene expression at several levels [2]. LncRNAs have been described in all taxa including plants, animals, prokaryotes, yeasts, and viruses [3] and their sequence conservation is usually lower than that of coding RNAs. Historically, they have been classified with respect to an arbitrary length size of more than 200 nucleotides and, according to their genomic location, are divided into four sub classes: long intergenic ncRNA (lincRNA), long antisense ncRNA, long sense overlapping ncRNA, and long sense intronic ncRNA [4]. The availability of robust machine learning methods for the identification of lncRNAs, which take into account the species-specific features, is crucial in the development of automatic annotation pipelines especially for less annotated genomes, such as zebrafish.

Several methods have been used to distinguish lncRNAs from other kinds of transcripts [5, 6], some of which are part of automatic annotation pipelines in Ensembl^1^ and UCSC^2^. For the purpose of this study, we separate methods into three main categories: i) feature-based classification tools, ii) coding potential detection tools, and iii) integrative pipelines for large scale annotation.

The first category includes tools based on a classifier trained with a set of features extracted from transcript sequences. The classifier is then used to predict new potential lncRNAs. The most relevant tools in this category are: *IseeRNA* – limited to the subclass of lincRNAs and is based on a Support Vector Machine classifier trained with conservation score, open reading frame length, and di/tri-nucleotide sequence frequencies [7]; *PLEK* – uses a Support Vector Machine trained with an improved k-mer scheme to distinguish lncRNAs from messenger RNAs (mRNAs) in the absence of genomic sequences or annotations [8]; *lncRNA-MFDL* – uses a deep learning algorithm with multiple features of the open reading frame, k-mer, secondary structure, and the most-like coding domain sequence [9]; and Lv et al. – uses LASSO regularization trained with genomic and chromatin features [10].

The second category of tools focuses on detecting the coding potential of a transcript and is generally used to discard coding transcripts in lncRNA identification pipelines. However, recently it has been demonstrated that transcripts previously classified as lncRNAs are indeed coding and represent a source of new peptides [11, 12]. The most prominent tools in this category are: *CPC* – evaluates the coding potential by using a Support Vector Machine trained with six biological features such as, BLAST similarity with known proteins, ORF length, and frame integrity [13]; *CPAT* – computes the coding potential with a logistic regression based on open reading frame and nucleotide arrangement metrics [14]; *PhyloCSF* – adopts a statistical phylogenetic codon models to evaluate whether a sequence is likely to represent a conserved protein coding region or not [15]; and *RNAcode* – relies on evolutionary signatures, including synonymous/conservative mutations and conservation of the reading frame, to predict protein coding regions in a set of homologous nucleotide sequences [16].

The third category includes pipelines supporting large scale analysis and annotation of novel lncNRAs in available genomes or in trascriptomes assembled from RNA-seq experiments. They integrate pre/post filtering steps with one or more of the approaches mentioned previously, in some cases, exploiting also other kind of data, such as ss expression level and histone modification. Cabili et al. produced a reference catalog of ∼ 8200 human lincRNAs using structural, expression, evolutionary features, and PhyloCSF to remove de novo assembled transcripts with high coding potential [17]. *Sebnif* uses IseeRNA and applies post filtering steps based on expression level data [18]. *Annocript* combines information of protein coding transcripts stored in genome databases to annotate novel lncRNAs in a whole transcriptome scale [19]. Li et al. use the Codon Substitution Frequency score to identify lincRNAs from de novo assembled transcripts in chicken skeletal muscle [20]. Pauli et al. use a pipeline based on PhyloCSF, ORF length, and protein homologs identified with BLASTP and HMMER to perform a large scale study of lncRNAs in zebrafish [21]. Ulitsky et al. use a filtering based pipeline to identify lincRNAs in zebrafish using 3P-seq, ChIP-seq, poly(A) sites, and H3K4me3 peaks [22]. Kaushik et al. use a pipeline to identify tissue specific lncRNAs in zebrafish based on ORF, coding potential, and protein Ref-Seq features [23].

In this study, we assemble several features used by the first category of tools to systematically evaluate their ability to recognize novel lncRNAs. We use different feature selection algorithms, test for feature stability, group features into signatures, and evaluate the prediction capability of a signature with a machine learning algorithm. We also include in the study a new category of genomic features based on repeats contained in transposable elements, motivated by the work of Jonson et al. [24]. Transposable elements represent the most abundant and functionally relevant class of repeats [25, 26] and it has been shown that non-coding genes, especially miRNAs and lncRNAs, are derived from these elements [27]. We show that such features are often selected by algorithms and each species seems to exhibit its own relevant sub-category of transposable elements. We show that there are different combinations of features that exhibit similar predictive performance. We collect them into signatures for three different species, human, mouse, and zebrafish, illustrating that some features are shared among species, while others are peculiar to a single species. The predictive performance of the obtained signatures compared with the current state of the art shows an improvement ranging from 1 to 24%, depending on the signature and on the species. The most significant improvement can be observed in zebrafish, the least annotated genome used in the study. This suggests that the method proposed in this paper has the potential to support the annotation of new and poorly characterized genomes in order to discover novel lncRNA candidates.

## Methods

### Genomic features

We collect 125 to 130 genomic features, depending on species, and grouped them into 5 different categories: Basic features (3 features), Open reading frame metrics (3 features), Conservation scores (3–6 features), Nucleotide compositions and arrangements (80 features). Moreover, we also use some novel features based on repeat elements (31–36 features). Additional file 1: Table S1 summarizes all the considered features.

- *Basic features (BASIC).* A transcript is defined as a single model annotated on a genome sequence with a set of coordinates that correspond to an exonic structure. We consider three features: the number of exons (TxNex), the transcript length (TxLen), and the mean exons’ length (TxExLenAvg).
- *Open reading frame (ORF).* Open reading frame is the portion of DNA that occurs between a start codon and a termination codon which has the potential to code for a protein. We consider three features in this category, i.e. ORF length (OrfLen), ORF proportion (OrfProp), and KOZAK motif score (KOZAK), which is an indicator of valid ORF [28]. We compute the ORF length with an approach similar to UCSC txCdsPredict utility. ORF proportion is computed dividing ORF length by transcript length and KOZAK motif score is computed with the consensus matrices proposed in Grzegorski et al. [29].
- *Conservation score (CONS).* Various studies report that lncRNAs are less conserved as compared to protein coding [3, 22]. We use two approaches to score the conservation level of each nucleotide, phastCons [30] and phyloP [31]. We rely on the conservation scores pre calculated by the UCSC database (https://genome.ucsc.edu). In particular, we use the following UCSC tracks: PhastCons and PhyloP 100 and 20 ways for Human (ph100, py100, ph20, and py20), PhastCons and PhyloP 60 ways for Mouse (ph60, py60), and PhastCons and PhyloP 8 ways for Zebrafish (ph8, py8). We average the scores among each exon sequence and take, for each transcript, the mean, the maximum, and the minimum among the averaged exon scores (eg. ph8m, ph8mx, ph8mn).
- *Nucleotide compositions and arrangements (NUCLEO).* Many studies like iSeeRNA [7], Sebnif [18], CPAT [14], RNAcon [32], and lncRNA-MFDL [9] have considered mono, di- and tri-nucleotide frequencies as important features for distinguishing ncRNA classes from protein coding. There are 16 di-nucleotide combinations and 64 tri-nucleotide combinations. We use the frequency compositions, i.e. occurrence divided by the transcript length, of these 80 different combinations to represent the nucleotide composition of a transcript. In addition, we use the Fickett score [33] which is reported as an important feature for distinguishing ncRNA from protein coding in CPAT [14]. Basically, the Fickett score measures the coding potential based on compositional bias between codon positions by estimating how asymmetric is the distribution of nucleotides at the three triplet positions in the sequence [34].
- *Repeat elements (REPS).* It has been shown that almost half of the human genome consists of repeated sequences (repeats), patterns of DNA or RNA that occur in multiple copies [25, 26]. Among these, transposable elements (TEs) represent the most abundant and functionally relevant class of repeats. It seems that non coding genes, especially miRNAs and lncRNAs, derive from transposable elements [24, 27]. In particular, lncRNAs are enriched in ∼ 83% of their sequence by TEs, against 39% of protein coding sequences [35]. As highlighted in the “RIDL hypothesis” [24], TEs act in lncRNA as functional binding domains and it seems that the presence of TEs allows lncRNA folding thermodynamically more stable. We consider transposable elements computed with the RepeatMasker tool available in the UCSC genome database. We consider only a subset of 81 relevant repeat families belonging to DNA and Rolling-circle transposons, LINE, SINE, LTR and Retrotransposons. Additional file 2: Table S2 reports all the collected families of repeats detected by RepeatMasker and summarizes for each family their relevance in each species. Each repeat family represents a feature that is computed for each transcript by considering its overlapping proportion within the transcript sequence.

### Feature selection and ranking

Feature selection is the process of identifying subsets of relevant features within a dataset [36]. The basic assumption is that data may contain redundant features. We filter out constant features, cluster together highly correlated features, and then we use feature selection algorithms to rank features according to their relevance.

#### Detecting highly correlated features

Multicollinearity refers to the non-independence of features so that the relationship of those features with the independent variables is distorted by the relationship between them. For prediction tasks, multicollinearity is not a problem as the predictions will still be accurate. Instead, in investigating which are the most important features in a classification problem, highly related features could compete for the same rank. We perform multicollinearity detection by computing the absolute Pearson correlation among all pairs of standardized features. Standardization, i.e. subtracting the mean and dividing by the standard deviation, of each feature is performed to avoid high correlation due to different scales of values. Then, we hierarchically cluster features by using the inverse absolute correlation distance and complete linkage. Clusters with a minimum intra absolute correlation greater than 0.8 are considered highly correlated clusters of features. Features belonging to a highly correlated cluster are replaced with a proxy feature chosen by those, in the cluster, that exhibit the highest univariate predictive value for the response class. To avoid dependence on small data perturbation, we use a hierarchical clustering strategy that assesses the uncertainty for each cluster via multiscale bootstrap re-sampling [37]. This technique allowed us to include only stable clusters of features, i.e. those that do not depend on small perturbation of data (*p*-value <0.05).

#### Multivariate feature ranking

We use 11 different feature selection approaches spanning three main categories [36, 38]: filter based, wrapper based, and embedded. We further add ensemble methods which have gained attention in several contexts [39, 40].

- *Filter based methods*, also known as univariate filter methods, rank all variables in terms of relevance, as measured by a score which depends on the method. A signature of size *k* can be obtained by taking the top *k* features according to the score. We consider Wilcox test (WT), Information Gain (IG), Gain Ratio (GR), and Relief Feature Elimination (RFS) [36].
- *Wrapper based methods* embed a classifier model hypothesis and attempt to jointly select sets of features with good predictive power for that classifier. We consider Recursive Feature Elimination (RFE) with a Support Vector Machine (SVM) classifier [36] and Greedy Forward Selection (GFS) with least squares regression [41]. In Recursive Feature Elimination algorithms, the worst feature is eliminated at each iteration so a signature of size *k* can be obtained by considering the last *k* eliminated features. Instead, in Greedy Forward Selection, at each iteration the best feature, i.e. the one which minimizes the sum of squares, is added to the model so a signature of size *k* can be obtained by considering the first *k* features added.
- *Embedded methods* search for an optimal subset of features during the training process of a classifier. We consider Lasso regression (LR) [42], Elastic Net (EN) [43], and Random Forest (RF) [44]. In Lasso regression and Elastic Net a signature of size *k* can be obtained by fixing *λ*, the parameter that controls the sparsity of a solution (i.e., the number of features selected), to the smallest value which gives a signature of *k* [45]. In Random Forest, the values for each feature are randomly shuffled and classified. The difference between the average margin of non-shuffled and shuffled instances provides a quality estimate of the attribute. The algorithm returns a scored list of features so a signature of fixed size *k* can be obtained by taking the top *k* features according to the score.
- *Ensemble methods* merge the outcomes of different algorithms so that the advantage of one algorithm could complete the weaknesses of another [46]. We aggregate the outcomes of *B* different feature selection algorithms by computing a score *S*_*f*_ for each feature *f* as an average function of its rank $r_{f}^{b}$ in the *b*-th experiment. We consider two functions of the rank for aggregation:
  1. *Ens-mean* (EFmn), average of the ranks of a feature over all outcomes, $S_{f} = 1/B \sum _{b=1}^{B} r_{f}^{b}$;
  2. *Ens-voting* (EFmd), mode of the ranks of a feature over all outcomes, $S_{f} = mode \left \{r_{f}^{b}\right \}_{b=1}^{B}$.

#### Feature stability

Feature selection methods are known to be sensitive to small perturbations of the training data, resulting in unstable signatures. This may affect the interpretation of results by focusing on features that have been selected just by chance. Some methods, such as Random Forest and Ensemble, embed strategies to reduce the dependence from small perturbations. We evaluate the stability of each signature in order to identify those that exhibit a greater stability. To assess the stability of a signature *S* of size *k*, we compare that signature with those estimated on different subsamplings of the training set {*S*1′,*S*2′,…,*S* *n*′}. We randomly subsample with 80% of sample overlap, estimate a signature of size *k* on each subset $S^{\prime }_{i}$, and compute the overlap between *S* and $S^{\prime }_{i}$ as the fraction of shared features, (*S*∩*S* *i*′)/*k*. The random sampling of subsets is repeated 100 times, and the stability values are averaged over all subsets. We also verify whether the stability of an algorithm can be improved with an ensemble procedure, so we aggregate the outcomes of a feature selection algorithm applied on *B* random subsamples of the training data (i.e., draw the 80% of samples with replacement *B* times) by using the two aggregation function introduced above.

#### Predictive accuracy of a signature

Feature selection algorithms that exhibit high stability rates do not guarantee that the generated signatures will also exhibit high discriminative capabilities. Thus we perform a set of experiments to evaluate the prediction capability of a signature adopting three different supervised machine learning algorithms: Support Vector Machine (SVM) [47] (Gaussian radial kernel and C =1), Random Forest (RForest) [44], and Naive Bayes (NBayes) [48]. Each algorithm is trained with signatures with an increasing number of features and is evaluated in a 10-fold cross validation scheme.

Predictions are compared against the gold standard described in the next section. We use Precision-Recall (PR) measures, in addition to the area under ROC curves (AUC), because they give a more informative picture of performance when dealing with highly skewed datasets [49]. In our case, the number of negative examples greatly exceeds the number of positives, therefore a large change in the number of false positives can lead to a small change in the false positive rate used in ROC analysis. In particular, we use a normalized version of the area under the PR curve (AUPR) that takes into account the unachievable region in PR space by using the method proposed in Boyd et al. [50]. This allows for comparing performance estimated on datasets with different class skews.

In addition to multivariate feature ranking, we evaluate also the ability, measured in terms of AUPR, of a single feature to correlate with the lncRNA class (univariate feature ranking).

The diversity in occurrence of each class in the training dataset can have a negative impact on model fitting. To avoid this problem, we apply to the training dataset the sampling approach proposed in ROSE [51] that down-samples the majority class and synthesizes new examples in the minority class.

### Comparison with other tools

To compare the prediction accuracy of a signature against state of the art methods, we consider two lncRNA prediction tools, IseeRNA and PLEK, and two coding potential detection tools, CPC and CPAT. As some of such tools (CPC and CPAT) are only available on-line, we perform a repeated (20 times) holdout validation by selecting, from the gold standard, a random test set of 100 transcripts in each class and evaluate the prediction accuracy of each tool. The same test set of transcripts is classified with a SVM classifier trained with signatures obtained with a training set where the used test set has been removed. The outcome predictions are evaluated in terms of Accuracy, Precision, and Recall.

### Gold–standard datasets

#### Annotated transcripts

We collect the annotated transcripts of three different species: human (hg38), mouse (mm10), and zebrafish (zv9/danRer7). Table 1 shows the distribution of collected transcripts, in Ensembl and Vega, among different categories, protein coding transcript (PCT), long ncRNA (lincRNA, intronic, overlapping, and antisense), pseudogene, other ncRNA, and IG/TR genes [52, 53]. The Pseudogene category includes transcripts predicted by the automated annotation procedure of Ensembl, while in the *other ncRNA* category we include: microRNA, piwi-interacting RNA, ribsosomal RNA, small interfering RNA, small nuclear RNA, small nucleolar RNA, transfer RNA, and vaultRNA. For the purpose of this study we consider only transcripts with reliable annotations – i.e. transcripts annotated in Vega (owning a Vega ID) and transcripts with a *KNOWN* status (reported in other external databases, such as Entrez and HGNC for human, MGI for mouse and ZFIN for zebrafish). Those selected reliable annotated transcripts are not necessary the outcome of typical automatic lncRNA annotation pipelines but are the result of manual inspections performed by the Havana group and are supported by strong experimental evidence. We filtered out pseudogenes because of their unstable annotation and divided the dataset in two classes: *lncRNA* (positive class) and *Other* (negative class), including in the latter category all reliable annotated transcripts that are not *lncRNA* (i.e. PCT + *other ncRNA* + *IG/TR genes*). The class skewness, i.e. the ratio between positives and negatives, is 0.18 (25821/139708) in human, 0.10 (8155/79486) in mouse, and 0.10 (1769/16754) in zebrafish.

**Table 1:** Distribution of different class of transcripts among Human, Mouse, and Zebrafish in Ensembl and Vega annotation databases

	Ensemble			Vega with KNOWN status		
Class	Human	Mouse	Zebrafish	Human	Mouse	Zebrafish
PCT	79851	50607	41695	71030	41569	11051
LincRNA	13473	5362	1039	13365	4711	1004
Intronic	977	277	58	973	277	57
Overlapping	343	47	9	342	45	9
Antisense	11186	3208	711	11141	3122	699
Pseudogene	14537	9442	261	14491	9066	199
Other ncRNA	78167	43609	11664	68265	37421	5703
IG/TR genes	434	642		413	496	
Total	198968	113194	55437	180020	96707	18722

#### De novo assembled and non-annotated transcripts

The recent study of Pauli et al. [21] identified 1133 multi-exonic lncRNAs from 56535 de novo transcripts assembled with cufflinks and sculpture from nine RNA-seq studies of embryogenesis and adult tissues in zebrafish (17 samples). As a case study we classify such transcripts by using a SVM classifier trained with different combination of features: all, zebrafish signatures (Table 5), and features used in IseeRNA [7]. Transcripts scored with a probability greater that 0.5 are considered new lncRNA candidates. We compare to which extent our prediction overlaps with the outcome of Pauli et al. study. To evaluate the likelihood of our prediction we perform two complementary analyses:

1. *Co-expression of predicted lncRNAs with their neighbor protein-coding genes*. Several studies suggest that some lncRNAs can act in *cis*, by affecting the expression of their neighbor protein-coding transcripts (PCT), and that the co-expression profile of lncRNAs versus their neighbor PCT is higher than the co-expression profile exhibited by PCT versus their neighbor PCT [4, 17, 54, 55]. For example, in the 16 Human Body Map tissues, the proportion of lncRNA and neighbor PCTs having a Spearman correlation greater than 0.9 is higher than the proportion obtained from a random sample of neighbor protein coding genes (7.1% vs. 3.9%) [4]. Furthermore, in mouse the expression profile of 5563 novel non-coding transcripts revealed a co-expression with their neighbor protein-coding genes that is on average higher than the co-expression exhibited by coding transcripts [55]. Given this assumption, we test if such a correlation pattern is also valid for the novel predicted lncRNAs in zebrafish. In particular, we test whether the absolute Spearman correlation computed between lncRNA–PCT is higher than the absolute Spearman correlation computed between PCT–PCT. Two genes are considered neighbors if their genomic distance is less than a given threshold measured in kb. To test for the optimal distance, we consider three genomic windows, 20, 30, and 40 kb. As a baseline comparison, we compute also the absolute Spearman correlation between two random non-neighbor protein-coding genes.
2. *Ribosome profiling of predicted lncRNAs compared with protein-coding RNAs*. Ribosome profiling gives an estimate of ribosome occupancy along transcripts by digesting RNA and sequencing the portion that is bound by 80S ribosomes [56]. When ribosome profiling is applied to protein-coding transcripts, a drastic drop in ribosome occupancy in 3’ UTR can be observed. Instead, such a drop is not observed for non-coding transcripts because, in such cases, translational termination should not occur [57, 58]. On this basis, Guttman et al. introduced a metric, Ribosome Release Score (RRS), to distinguish between coding and non-coding transcripts showing a great separation between known protein-coding RNAs and known non-coding RNAs in mouse [58]. We use the same metric (RRS) to indirectly evaluate the authenticity of predicted lncRNAs in zebrafish by testing whether a significant RRS difference with protein-coding transcripts can be observed. For this, we use the GWIPS-viz database (http://gwips.ucc.ie), which provides on-line tools for the analysis, visualization, and download of a wide collection of ribo-seq data obtained with the ribosome profiling technique [59].

## Results and discussions

**Fig. 1:**
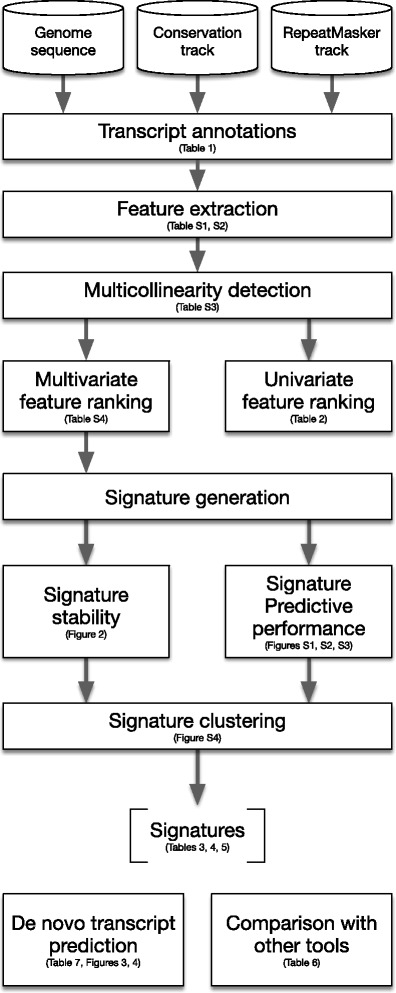
Analysis workflow. The analysis workflow adopted to obtain the signatures

In this section, we report the results obtained by applying the analysis workflow depicted in Fig. 1. All analyses can be reproduced by using the R-scripts available as Additional file 3.

### Multicollinear features

Additional file 4: Table S3 shows the detected clusters of highly correlated features. Some of them demonstrate obvious associations, such as: transcript length (TxLen) and ORF length (OrfLen), conservation scores computed with alternative tools (PhyloP and PhasCons), and di-/tri-nucleotides encoding similar information (TT vs TTT, GG vs GGG, CC vs CCC, AA vs AAA, GC vs GCC, TA vs ATA/TAT, GA vs AGA). Others refer to classes of repeats grouped among species in a different way. Some of these clusters have a clear biological interpretation. In zebrafish and mouse, non-autonomous repeats follow autonomous repeats (clusters DNA.P, LINE.RTE and DNA.DNA, LINE.RTE.X, respectively). This is required for the codification of enzymes necessary to the mechanism of transposition [60, 61]. In human, the only cluster related to transposable elements (DNA.hAT.Tag1, DNA.Merlin, DNA.TcMar) refers to hAT, Merlin and Tc1/Mariner superfamilies which belong to the Subclass I according to the Transponable Element classification and share the same “cut and paste" mechanism of genomic insertion [62]. Similarly, in mouse, the transposable element cluster (DNA.PiggyBac, LINE.Dong.R4 and RC.Helitron) includes superfamilies descending probably from the same ancestral transponable element called “Ancestral Vertebrate Mobilome" [63], suggesting a common evolutionary origin.

### Univariate feature ranking

Table 2 shows, for each species, the top 25 features ordered by AUPR. An overall performance decrement from human to mouse and then to zebrafish can be observed. The overall low performance in zebrafish may be related with lower annotation quality of its genome. In each species, conservation score related features (PhyloP and PhasCons) are the top most predictive features exhibiting an AUPR ranging between 0.43–0.62 in human, 0.25–0.43 in mouse, and 0.25–0.27 in zebrafish. This confirms that sequence conservation of lncRNAs is a peculiar characteristic. Transcript length related features (TxLen and TxNEx) are more predictive in human and zebrafish than in mouse.

**Table 2:** Univariate ranked features according to their AUPR (AUC)

	Human		Mouse		Zebrafish	
	Feature	AUPR (AUC)	Feature	AUPR (AUC)	Feature	AUPR (AUC)
1	ph100m	0.62 (0.92)	phm	0.43 (0.90)	py8m	0.27 (0.83)
2	ph20m	0.54 (0.91)	py60m	0.36 (0.90)	ph8m	0.25 (0.79)
3	ph20mx	0.52 (0.89)	phmx	0.34 (0.87)	TxLen	0.18 (0.72)
4	py100mx	0.52 (0.91)	py60mx	0.32 (0.88)	FickScore	0.17 (0.73)
5	py100m	0.48 (0.91)	phmn	0.25 (0.81)	TxNex	0.16 (0.77)
6	py20m	0.43 (0.89)	CG	0.16 (0.70)	GG	0.15 (0.66)
7	TxNex	0.26 (0.76)	GCG	0.15 (0.68)	TAA	0.15 (0.67)
8	ph20mn	0.25 (0.77)	CGC	0.14 (0.67)	AAT	0.15 (0.65)
9	CG	0.24 (0.69)	CGA	0.14 (0.67)	GAG	0.15 (0.65)
10	FickScore	0.23 (0.76)	CCG	0.13 (0.67)	GGA	0.14 (0.65)
11	CGA	0.22 (0.68)	CGG	0.13 (0.68)	KOZAK	0.14 (0.67)
12	TCG	0.21 (0.66)	ACA	0.13 (0.63)	GGC	0.13 (0.65)
13	CCG	0.21 (0.67)	FickScore	0.13 (0.73)	TCG	0.13 (0.63)
14	TxLen	0.19 (0.66)	TCG	0.13 (0.65)	ATT	0.13 (0.63)
15	KOZAK	0.17 (0.65)	CGT	0.12 (0.63)	CG	0.13 (0.62)
16	CGT	0.17 (0.62)	GC	0.12 (0.65)	TTG	0.13 (0.59)
17	ACA	0.17 (0.60)	CAT	0.12 (0.59)	TGG	0.13 (0.64)
18	ACG	0.17 (0.63)	ACG	0.12 (0.64)	CGG	0.13 (0.63)
19	ACT	0.16 (0.60)	ACT	0.12 (0.61)	CGA	0.13 (0.62)
20	TCT	0.16 (0.61)	GGC	0.11 (0.64)	CCG	0.12 (0.62)
21	TGG	0.15 (0.61)	TxNex	0.11 (0.73)	TT	0.12 (0.61)
22	AAT	0.15 (0.63)	KOZAK	0.10 (0.65)	TA	0.12 (0.62)
23	GTG	0.15 (0.60)	CTA	0.10 (0.59)	AG	0.12 (0.60)
24	GG	0.15 (0.62)	TxLen	0.10 (0.64)	AT	0.12 (0.62)
25	ATA	0.15 (0.61)	AC	0.09 (0.59)	CAG	0.12 (0.58)

Among other features, some tri-nucleotides occur in top ranks for all three species. Such tri-nucleotides seem to play a relevant role in the maintenance of secondary structure stability [64]. Many of them, like ACG, CCG, CGA, CGG, CGT, GCG, TAA, TAC, TCG and TAG, have been found in related studies for the classification of lincRNAs [7, 32], reflecting their importance for biological functions based on stable secondary structure. Furthermore, KOZAK and Fickett score features are top-ranked in all species, underscoring that the absence of the KOZAK motif, known to be associated with efficient translation [65] and lower coding potential based on nucleotide composition [33], are particularly important for identifying long non-coding transcripts.

### Multivariate feature ranking

Multiple features grouped together into a *signature* could improve the prediction performance of single features shown in Table 2. We compute the performance in terms of AUPR increasing the size of the signature for different feature selection algorithms and machine learning algorithms. The complete results of such experiments are shown in Additional files 5, 6 and 7, respectively for human, mouse, and zebrafish. For SVM and RForest, the maximum performance is almost asymptotically reached with a signature size ranging from 10 to 20 in all species, meaning that the first 10–20 features are the most informative. NBayes performance is almost constant or increases in the first top 20 features and then decreases, sometimes drastically. This has already been described in the literature: the NBayes classifier requires a number of samples that is logarithmic in the number of features, then at some point adding good features decreases test accuracy [48]. Additional file 8: Table S4 reports the complete list of features ranked by each algorithm for each species.

**Fig. 2:**
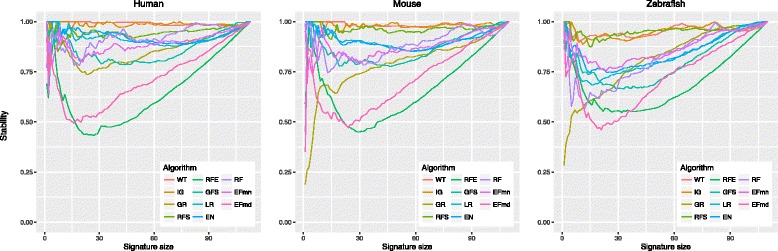
Signature stability. Stability of signatures averaged among 100 bootstraps for each feature selection algorithm (average stability on y-axis)

To identify the most significant signatures, we evaluated signature stability, chose signature size, and clustered similar signatures by using Jaccard distance. Figure 2 shows signature stability at different signature size for each feature selection algorithm, and for each species. The stability of almost all algorithms becomes rapidly more than 0.7, used as a threshold. We fix the size of a signature to 20, including the 20 top most informative features according to each algorithm. To detect the best signature overlap, we clustered the top 20 features of each algorithm with hierarchical clustering using Jaccard distance and complete linkage. Not all algorithms have been considered for clustering. RFE and EFmd were discarded because they were very unstable in all species and, for zebrafish, we discarded also GR, RF, and GFS because their stability is below 0.7 for signatures of size around 20. Additional file 9: Figure S4 shows the obtained signature clusters for each species. We group together signatures having at least 55% of features in common, cutting the hierarchical cluster tree at 0.45 and thus obtaining 5 signatures in human, 6 in mouse, and 4 in zebrafish as shown respectively in Tables 3, 4 and 5.

**Table 3:** Signatures detected in top 20 ranked features (Human)

Signatue #	Algorithm groups	BASIC	CONS	NUCLEO	ORF	REPS	AUPR (AUC)
1	IG, RFS,	TxExLenAvg,	ph100m,	AA, AAT, AT,	KOZAK,	DNA.TcMar.Tigger,	0.69 (0.94)
	RF,	TxLen,	ph20m,	ATA, CA, CC,	OrfProp	LINE.L1,	
	EFmn	TxNex	ph20mn,	CCG, CG,		LTR.ERV1,	
			ph20mx,	CGA, CGT,		LTR.ERVL,	
			py100m,	FickScore, GC,		LTR.ERVL.MaLR,	
			py100mx,	GG, GT, GTG,		SINE.Alu,	
			py20m	TA, TAT, TCG,		SINE.MIR	
				TT, TTA			
2	GR	TxExLenAvg	ph100m,	ATC, ATG, CA,		DNA.DNA,	0.55 (0.92)
			ph20m,	CAC		DNA.hAT.Blackjack,	
			ph20mx,			DNA.MULE.MuDR,	
			py100m,			DNA.PiggyBac,	
			py100mx,			DNA.TcMar.Tc2,	
			py20m			LINE.Penelope,	
						LTR.LTR,	
						RC.Helitron,	
						SINE.MIR	
3	GFS	TxExLenAvg,	ph100m,	AA, ACC, CA,	KOZAK	LINE.Penelope	0.67 (0.94)
		TxLen,	ph20mx,	CAG, CTA,			
		TxNex	py100m,	FickScore,			
			py20m	GAT, GT,			
				TAC, TAT,			
				TGG			
4	LR, EN	TxLen,	ph100m,	AA, AAT, ACA,	KOZAK		0.66 (0.94)
		TxNex	ph20m,	ACT, CA,			
			ph20mx,	CAA, CAC,			
			py100m,	CG, CGA,			
			py100mx	FickScore, GG,			
				GT, GTG,			
				TAC, TCT,			
				TGA, TGG			
5	5 WT	TxExLenAvg,		AAC, AAG,			0.66 (0.94)
		TxNex		AC, ACA,			
				ACC, ACG,			
				ACT, AGA,			
				AGC, AGT,			
				ATA, CA, CT,			
				GA, GT, TA,			
				TC, TG

**Table 4:** Signatures detected in top 20 ranked features (Mouse)

Signatue #	Algorithm groups	BASIC	CONS	NUCLEO	ORF	REPS	AUPR (AUC)
1	IG	TxNex	phm, phmn,	ACA, ACG,	KOZAK		0.47 (0.92)
			phmx,	CCG, CG,			
			py60m,	CGA, CGC,			
			py60mx	CGG, CGT,			
				FickScore, GC,			
				GCG, TAA,			
				TCG			
2	GR		phm, phmn,	ACA, AGA,		DNA.hAT.Charlie,	0.40 (0.91)
			phmx,	AT, CA, CAA,		LINE.RTE.BovB,	
			py60m,	CAT, CG, TGA		LINE.RTE.X,	
			py60mx			LTR.ERVL.MaLR,	
						SINE.ID,	
						SINE.MIR,	
						SINE.tRNA	
3	RFS	TxExLenAvg,	phm, phmn,	AA, FickScore	KOZAK,	LINE.L1,	0.44 (0.92)
		TxLen,	phmx,		OrfProp	LTR.ERV1,	
		TxNex	py60m,			LTR.ERVK,	
			py60mx			LTR.ERVL,	
						LTR.ERVL.MaLR,	
						SINE.Alu,	
						SINE.B2, SINE.B4	
4	GFS, LR,	TxExLenAvg,	phm, phmn,	AAC, AAG,	KOZAK		0.51 (0.93)
	EN	TxLen,	phmx,	AC, ACA, ACT,			
		TxNex	py60m,	AGT, CAC,			
			py60mx	CAG, CAT,			
				CGT, CTT,			
				FickScore,			
				GAT, GT,			
				GTA, GTC,			
				GTG, TAA,			
				TAC, TAT			
5	RF,	TxExLenAvg,	phm, phmn,	AA, AC,	KOZAK		0.51 (0.93)
	EFmn	TxLen,	phmx,	ACA, AGA,			
		TxNex	py60m,	CAC, CAT,			
			py60mx	CCG, CG,			
				CGC, CGG,			
				FickScore, GC,			
				GGC, GT,			
				TAA, TAT, TT			
6	WT	TxExLenAvg,	AAC, AAG,				0.46 (0.92)
		TxNex		AAT, AC,			
				ACA, ACC,			
				ACG, ACT,			
				AGA, AGC,			
				AT, CA, CG,			
				CT, GT, TA,			
				TC, TG			

Each signature exhibits a AUPR prediction performance ranging between 0.55–0.69 in human, 0.40–0.51 in mouse, and 0.32–0.41 in zebrafish. Compared to single feature performance reported in Table 2, the predictive performance obtained with group of features is higher, making the feature selection strategy the most effective for the classification of lncRNAs. This is evident especially in zebrafish (*Signature 3*) where the performance in terms of AUPR is almost twice that of the top univariate ranked feature, *py8m* (0.41 vs 0.27). In all species, features related with transcript length and conservation score are recurrent in almost all signatures. This basically confirms what is currently known in literature: lncRNA sequences are less conserved than protein-coding genes, but more than introns or random intergenic regions [3, 22, 66, 67]. ORF related features (KOZAK and OrfProp) are also included in almost all signatures. They probably take into account the low coding potential of lncRNAs. In some signatures, the Fickett score feature is selected in conjunction with other di-/tri-nucleotides features, while in others appears alone. In the first case no repeat features are selected, while in the latter a group of repeat features are selected as an alternative. Di- and tri-nucleotides considered in IseeRNA [7, 32] are also present in our signatures. Their presence together with repeat features captures the ability of a sequence to maintain a stable RNA structure [64], which is crucial for the functioning of lncRNAs. Di-/tri-nucleotides and repeats rarely appear together, and in most cases are mutually excluded. We argue that this selection denotes similar information contents. Another consideration about repeats is that some of them, such as LTR-ERVL/K, are specific to human and mouse, while others, for example LTR-DIRS, are found only in zebrafish. Similarly, DNA transposons are more enriched in zebrafish (75%) than in human and mouse (10%) [68], instead, LINEs and SINEs are more predominant in human and mouse than in zebrafish [60]. This could explain why in human and mouse we see signatures containing LINE/SINE and in zebrafish signatures containing DNA transposons.

### Comparison with other tools

As a baseline comparison, we computed AUPR and AUC performances obtained with IseeRNA, PLEK, CPC, and CPAT. For IseeRNA, we used a SVM classifier trained with the same features (PhastCons conservation score, ORF length and proportion, and frequencies of GC, CT, TAG, TGT, ACG, TCG) and the same settings reported in the original paper [7]. For PLEK, we used the available Python tool based on an improved k-mer scheme. For CPC and CPAT, we used the available web tools with default settings (respectively http://cpc.cbi.pku.edu.cn and http://lilab.research.bcm.edu/cpat).

Table 6 shows the results obtained in these experiments. Coding/non-coding tools (CPAT and CPC) and PLEX are outperformed by supervised approaches in terms of accuracy. The improvement ranges from 16 to 21% in human, from 13 to 24% in mouse, and from 12 to 23% in zebrafish. The signature of IseeRNA is moderately outperformed by SVM signatures in zebrafish (8% for *Signature 3*). Instead, in human and mouse, the performances of IseeRNA and SVM signatures are comparable.

**Table 5:** Signatures detected in top 20 ranked features (Zebrafish)

Signatue #	Algorithm groups	BASIC	CONS	NUCLEO	ORF	REPS	AUPR (AUC)
1	IG	TxExLenAvg,	ph8m, py8m,	AAT, ACG,	KOZAK,		0.39 (0.90)
		TxLen,	py8mn	ATT, CCG,	OrfProp		
		TxNex		CG, CGA,			
				CGC, CGG,			
				FickScore,			
				GAG, GG,			
				GGA, GGC,			
				TA, TAA,			
				TCG, TGG,			
				TT, TTG			
2	RFS	TxExLenAvg,	ph8m, py8m,	FickScore	KOZAK,	DNA.DNA,	0.32 (0.87)
		TxLen,	py8mn		OrfProp	DNA.hAT,	
		TxNex				DNA.hAT.Ac,	
						DNA.hAT.Charlie,	
						DNA.hAT.Tip100,	
						DNA.Kolobok,	
						DNA.PiggyBac,	
						DNA.TcMar.Tc1,	
						LINE.L2, SINE.5S,	
						SINE.V	
3	LR, EN,	TxExLenAvg,	ph8m, py8m,	AA, AAT, ACA,	KOZAK,		0.41 (0.90)
	EFmn	TxLen,	py8mn	ACT, AGT,	OrfProp		
		TxNex		CAT, CGC,			
				CTA, CTC,			
				FickScore,			
				GAG, GC,			
				GCC, GGA,			
				TAA, TAC,			
				TCC, TGA,			
				TGG, TTG			
4	WT	TxNex		AAC, AAT,			0.36 (0.89)
				ACA, ACC,			
				ACG, AG,			
				AGC, AGG,			
				AT, CAG,			
				CCA, CCG,			
				CG, CGA,			
				CG, CGA,			
				GG, TC

**Table 6:** Performance of tested tools (average Precision/Recall/Accuracy with 95% CI)

	Coding/non-coding tools				SVM						
	CPAT	CPC	PLEK	iSeeRNA	All features	Signature 1	Signature 2	Signature 3	Signature 4	Signature 5	Signature 6
human											
Precision	0.71 (± 0.02)	0.81 (± 0.02)	0.67 (± 0.01)	0.97 (± 0.01)	0.97 (± 0.01)	0.97 (± 0.01)	0.97 (± 0.01)	0.96 (± 0.01)	0.97 (± 0.01)	0.98 (± 0.02)	−
Recall	0.96 (± 0.01)	0.66 (± 0.03)	0.98 (± 0.01)	0.91 (± 0.02)	0.93 (± 0.01)	0.94 (± 0.02)	0.91 (± 0.02)	0.94 (± 0.01)	0.93 (± 0.01)	0.82 (± 0.04)	−
Accuracy	0.78 (± 0.01)	0.75 (± 0.02)	0.74 (± 0.01)	0.94 (± 0.01)	0.95 (± 0.01)	0.95 (± 0.01)	0.94 (± 0.01)	0.95 (± 0.01)	0.95 (± 0.01)	0.90 (± 0.02)	−
mouse											
Precision	0.73 (± 0.01)	0.84 (± 0.02)	0.66 (± 0.01)	0.99 (± 0.01)	0.99 (± 0.01)	0.99 (± 0.01)	0.99 (± 0.01)	0.99 (± 0.01)	0.99 (± 0.01)	0.99 (± 0.01)	0.99 (± 0.01)
Recall	0.96 (± 0.01)	0.77 (± 0.02)	0.91 (± 0.02)	0.87 (± 0.02)	0.91 (± 0.02)	0.90 (± 0.01)	0.88 (± 0.02)	0.90 (± 0.02)	0.91 (± 0.02)	0.91 (± 0.01)	0.82 (± 0.07)
Accuracy	0.81 (± 0.01)	0.81 (± 0.01)	0.71 (± 0.02)	0.93 (± 0.01)	0.95 (± 0.02)	0.94 (± 0.01)	0.94 (± 0.01)	0.94 (± 0.01)	0.95 (± 0.01)	0.95 (± 0.01)	0.90 (± 0.04)
zebrafish											
Precision	0.83 (± 0.01)	0.85 (± 0.01)	0.72 (± 0.01)	1.00 (± 0.01)	1.00 (± 0.01)	1.00 (± 0.01)	1.00 (± 0.00)	1.00 (± 0.00)	1.00 (± 0.01)	−	−
Recall	0.86 (± 0.03)	0.74 (± 0.03)	0.82 (± 0.04)	0.86 (± 0.03)	0.91 (± 0.01)	0.91 (± 0.02)	0.90 (± 0.02)	0.96 (± 0.02)	0.80 (± 0.06)	−	−
Accuracy	0.84 (± 0.02)	0.81 (± 0.02)	0.74 (± 0.02)	0.89 (± 0.02)	0.94 (± 0.01)	0.93 (± 0.01)	0.93 (± 0.01)	0.97 (± 0.02)	0.85 (± 0.04)	−	−

### Case study: prediction of novel lncRNAs in zebrafish

As a case study, we collected 56535 new zebrafish transcripts assembled from RNA-seq experiments in the study of Pauli et al. [21] who identified 1133 putative lncRNAs. The pipeline basically filters out transcripts with high coding potential estimated with PhyloCSF, high ORF quality, and known protein homologs estimated with blastx, blastp, and HMMER. We classified the same set of transcripts by using a SVM classifier trained with the set of annotated zebrafish transcripts (Table 1). Table 7 summarizes the results obtained considering different combination of features: all, zebrafish signatures (Table 5), and features used in IseeRNA [7]. The overlap with Pauli et al. predictions reaches the maximum with *Signature 3* (92%) which is not far from *Signature 1* and *Signature 2* (88 and 91%) and a little more greater than *Signature 4* and the IseeRNA signature (85 and 84%). Using all features reduces the fraction to 65%.

**Table 7:** Pauli et al. [21] novel transcripts predicted with different zebrafish signatures

Training Features	Predicted lncRNAs	Pauli et al. lncRNAs	Intersection	Fraction	AUC
Signature 1	17154	1133	1035	0.91	0.87
Signature 2	17305	1133	995	0.88	0.87
Signature 3	17198	1133	1039	0.92	0.87
Signature 4	18615	1133	962	0.85	0.81
IseeRNA	17077	1133	951	0.84	0.82
All	9366	1133	738	0.65	0.78

To verify the bona fides from our putative lncRNAs, we followed two strategies: 1) co-expression of predicted lncRNAs with their neighbor protein-coding genes; and 2) ribosome profiling of predicted lncRNAs compared with protein-coding RNAs.

**Fig. 3:**
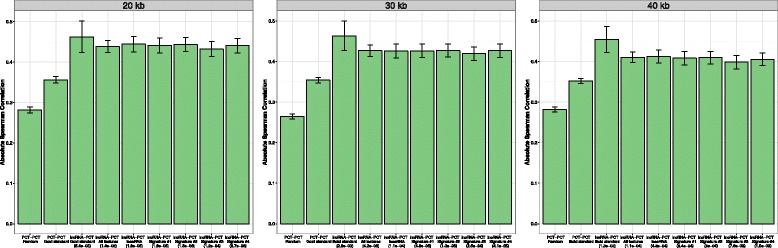
Co-expression with neighbor protein coding genes evaluated for transcripts classified with different zebrafish signatures. Co-expression with neighbor protein coding genes is evaluated with the absolute Spearman correlation for transcripts classified with different zebrafish signatures and at different kb windows. In parentheses the *p*value of one tailed wilcox test between lncRNAs–PCT and PCT-PCT (Gold-standard) distributions

In the first analysis, we tested whether our putative lncRNAs follow a co–expression profile with PCT neighbors similar to that observed in other studies [4, 55]. We collected an expression dataset of 17 samples provided in Pauli et al. [21] representing 8 time-points of zebrafish embryo-genesis stages (Accession numbers: PRJNA154389, GSE32898) and mapped the predicted transcripts using TopHat and Cufflinks pipelines [69]. We filtered out 50% of transcripts with low expression variation among all the samples, obtaining an expression matrix of 11015 transcripts in 17 samples. We computed the absolute Spearman correlation between the top 10% of predicted lncRNAs and neighbor annotated PCTs at different kb windows. Figure 3 shows the absolute Spearman correlation of lncRNA–PCT pairs, a sample of annotated PCT–PCT pairs, and a sample of random not neighbor PCT–PCT pairs for each considered window. In all cases, lncRNA–PCT pairs exhibit a higher correlation with respect to annotated PCT–PCT pairs (statistical significance tested with one tailed wilcox test and shown in parentheses). No significant difference is found among signatures even for the gold standard set. An overall increase in significance is observed at 20 kb window, similar to that reported in other related studies [4, 55].

**Fig. 4:**
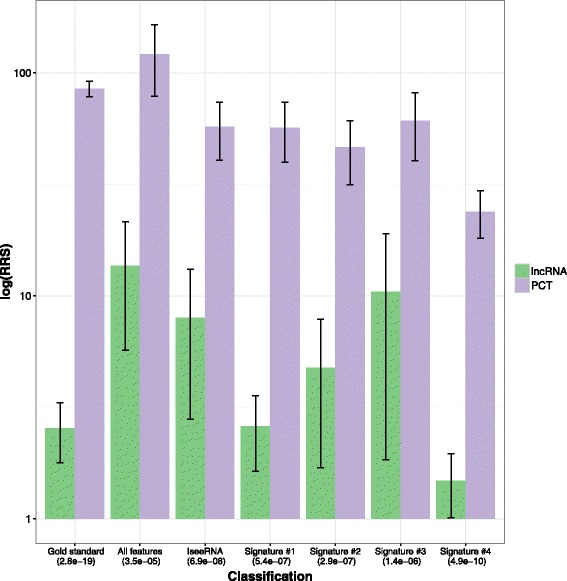
Ribosome Release Score evaluated for transcripts classified with different zebrafish signatures. The Ribosome Release Score (RRS), a relative measure of abundance of ribosomes reads in ORF and 3’UTR regions, is evaluated for transcripts classified with different zebrafish signatures and for those belonging to the gold standard (Table 1). In parentheses the *p*value of one tailed wilcox test between PCTs and lncRNAs distributions

In the second analysis, we tested whether our putative lncRNAs exhibit a Ribosome Release Score (RRS) significantly lower than protein-coding RNAs [58]. We collected the zebrafish ribo-seq profile provided by GWIPS-viz database, which is an aggregate of two ribo-seq studies [70, 71], and the mRNA-seq profile provided by Pauli et al. [21]. We computed the RRS of the top 10% lncRNAs predicted with different combination of features and those belonging to the zebrafish gold standard (Table 1). As shown in Fig. 4, in all cases the RRS of protein-coding RNAs is greater than the RRS of lncRNAs (statistical significance tested with one tailed wilcox test and shown in parentheses). As expected, the most significant difference can be observed for annotated lncRNAs (*p*value ≤2.8·10^−19^). *Signature 4* exhibits the most significant difference (*p*value ≤4.9·10^−10^).

## Conclusions

LncRNA peculiarities, such as transcripts length and poor conservation at primary sequence level between species, pose a variety of new computational biology challenges: identification of novel lncRNA genes, and understanding how they evolve and function. Large scale studies on human, mouse, and zebrafish, for which a large number of genomic, transcriptomic and expression data are available, are instrumental for comparative analyses aimed at: 1) developing lncRNA discovery tools that produce a high-quality set of lncRNAs from RNA-seq data; 2) allowing comprehensive annotation of lncRNAs with respect to their primary sequences, the structural features, and their related functions; 3) searching for signatures and features that help to find common codes, even at the level of short nucleotide sequences, used by lncRNA in the course of evolution; and 4) elucidating evolutionary constraints in order to prioritize which lncRNAs that are likely to be functionally important.

We performed an extensive comparison of a number of features extracted from transcript sequences. Some of them were borrowed from literature and others, related to repeats, were novel additions. With the proposed analysis, we obtained different signatures for human, mouse, and zebrafish, highlighting features are shared among species, while identifying those peculiar to a single species. All signatures obtained in this study outperform the prediction performance reported in the literature by 1–24% depending on the signature and species, showing that the systematic selection of informative features could improve classification performance.

With the obtained signatures, we classified 56535 de novo assembled transcripts of zebrafish and validated the obtained putative lncRNAs with two in-silico strategies: 1) co-expression pattern with respect to neighbor protein-coding genes, and 2) ribosome profiling compared with protein-coding RNAs. Both analyses revealed a significant enrichment for predicted lncRNAs with respect to protein-coding genes corroborating the likelihood of our predictions.

Studies including more animal species are needed to fully generalize our results, nonetheless we have shown that our methodology can be easily extended to include additional features – not necessary extracted from sequences – and applied to other genomes.

## Endnotes

^1^ http://www.ensembl.org

^2^ https://genome.ucsc.edu

## Additional files

Additional file 1 **Table S1.** Features considered in the study. (XLS 46 kb)

Additional file 2 **Table S2.** Collected families of repeats. (XLS 2220 kb)

Additional file 3 **R scripts.** R scripts adopted for the experiments described in the paper. (ZIP 44 kb)

Additional file 4 **Table S3.** Clusters of highly correlated features. (PDF 31 kb)

Additional file 5 **Figure S1.** Prediction performance in terms of AUPR for different feature selection algorithms at increasing size of the signature in human. (PDF 72 kb)

Additional file 6 **Figure S2.** Prediction performance in terms of AUPR for different feature selection algorithms at increasing size of the signature in mouse. (PDF 72 kb)

Additional file 7 **Figure S3.** Prediction performance in terms of AUPR for different feature selection algorithms at increasing size of the signature in zebrafish. (PDF 72 kb)

Additional file 8 **Table S4.** List of features ranked by each algorithm in each species. (XLS 63 kb)

Additional file 9 **Figure S4.** Hierarchical clusters of top 20 features selected by different algorithms computed with Jaccard distance, i.e. ratio between intersection and union of two sets, and complete linkage.(PDF 37 kb)

## Abbreviations

AUC: Area under the ROC curve
AUPR: Area under the precision recall curve
lncRNA: Long non-coding RNA
PCT: Protein coding transcript
ROC: Receiver operating characteristic
RRS: Ribosome release score
