# Supplementary material for: Identification of long non-coding transcripts with feature selection: a comparative study
Source: BMC Bioinformatics. 2017 Mar 23;18:187. doi: 10.1186/s12859-017-1594-z (PMC5364679; doi:10.1186/s12859-017-1594-z)
Supplement: Supplementary file 4 — Table S3. Clusters of highly correlated features. (PDF 31 kb) [file 12859_2017_1594_MOESM4_ESM.pdf]

TopFeature	Cluster	Cohesion
	Human	
DNA.hAT.Tag1	DNA.hAT.Tag1, DNA.Merlin, DNA.TcMar	1.00
TxLen	TxLen, OrfLen	1.00
AA	AA, AAA	0.95
TT	TT, TTT	0.95
CC	CC, CCC	0.94
GG	GG, GGG	0.91
ph20mx	ph20mx, ph100mx, py20mx	0.90
AT	AT, ATT	0.87
GC	GC, GCC	0.87
CCG	CCG, CGC	0.86
CG	CG, CGG, GCG	0.85
AAT	AAT, TAA	0.83
ph20mn	ph20mn, ph100mn, py20mn, py100mn	0.80
	Mouse	
RC.Helitron	DNA.PiggyBac, LINE.Dong.R4, RC.Helitron	1.00
TxLen	TxLen, OrfLen	1.00
TT	TT, TTT	0.95
AA	AA, AAA	0.94
CC	CC, CCC	0.92
phmn	phmn, py60mn	0.90
LINE.RTE.X	DNA.DNA, LINE.RTE.X	0.88
GG	GG, GGG	0.88
GC	GC, GCC	0.82
LINE.RTE.BovB	DNA.hAT, DNA.MULE.MuDR, LINE.RTE.BovB, LTR.LTR	0.81
	Zebrafish	
TxLen	TxLen, OrfLen	1.00
TT	TT, TTT	0.95
DNA.P	DNA.P, LINE.RTE	0.94
AA	AA, AAA	0.93
py8m	py8m, py8mx	0.90
CC	CC, CCC	0.86
py8mn	ph8mn, py8mn	0.83
LINE.I	LINE.I, LTR.LTR	0.82
GA	GA, AGA	0.81
ATT	ATT, TTA	0.80
ph8m	ph8m, ph8mx	0.80
TA	TA, ATA, TAT	0.80

Table S4. Detected multicollinear clusters of features
