# Supplementary material for: Identification of long non-coding transcripts with feature selection: a comparative study
Source: BMC Bioinformatics. 2017 Mar 23;18:187. doi: 10.1186/s12859-017-1594-z (PMC5364679; doi:10.1186/s12859-017-1594-z)
Supplement: Supplementary file 5 — Figure S1. Prediction performance in terms of AUPR for different feature selection algorithms at increasing size of the signature in human. (PDF 72 kb) [file 12859_2017_1594_MOESM5_ESM.pdf]

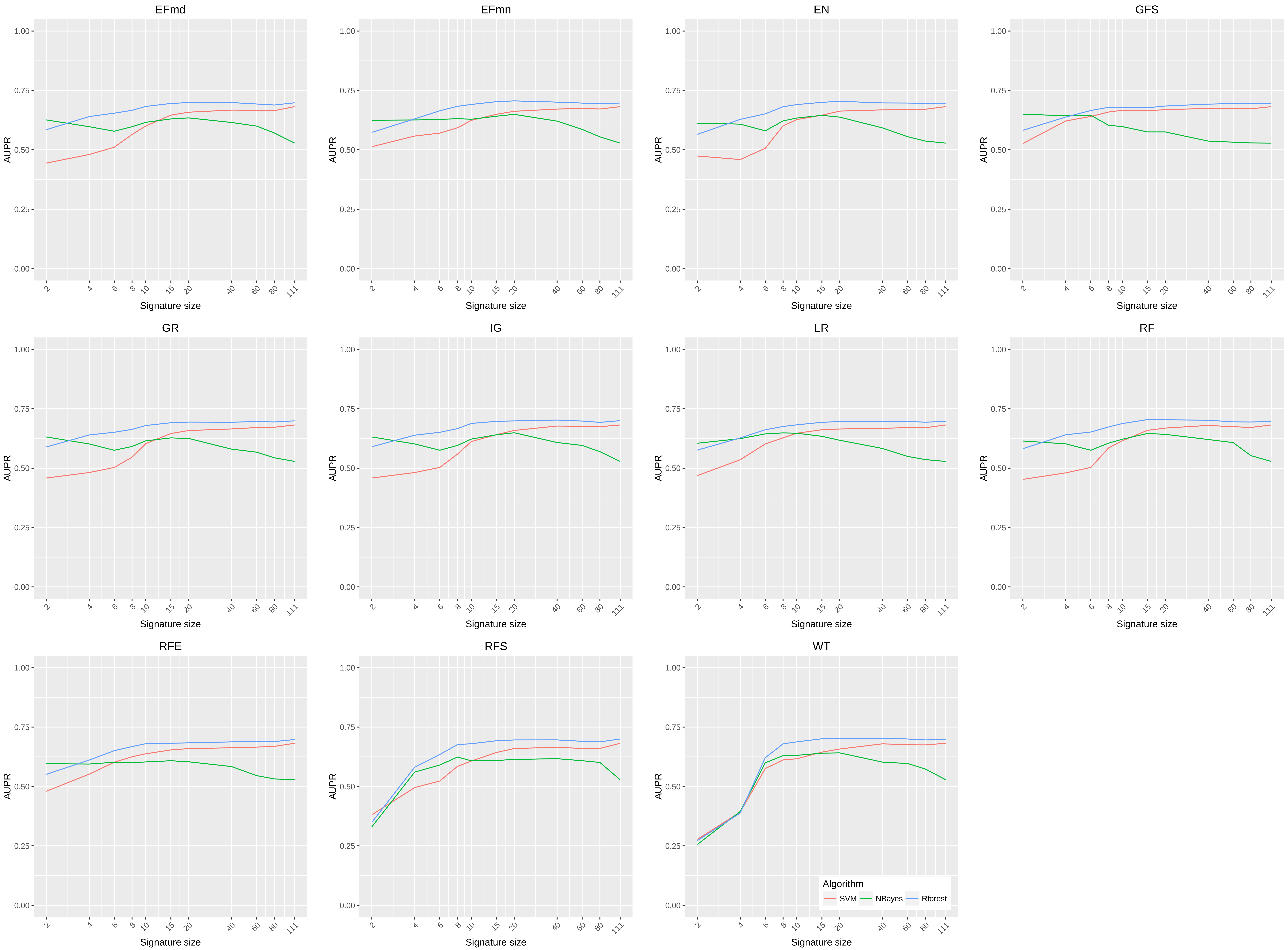

Figure S1. Prediction accuracy with different feature selection algorithms in human (10-fold average AUPR on the y-axis, signature size on the x-axis)
