# Supplementary material for: Identification of long non-coding transcripts with feature selection: a comparative study
Source: BMC Bioinformatics. 2017 Mar 23;18:187. doi: 10.1186/s12859-017-1594-z (PMC5364679; doi:10.1186/s12859-017-1594-z)
Supplement: Supplementary file 9 — Figure S4. Hierarchical clusters of top 20 features selected by different algorithms computed with Jaccard distance, i.e. ratio between intersection and union of two sets, and complete linkage.(PDF 37 kb) [file 12859_2017_1594_MOESM9_ESM.pdf]

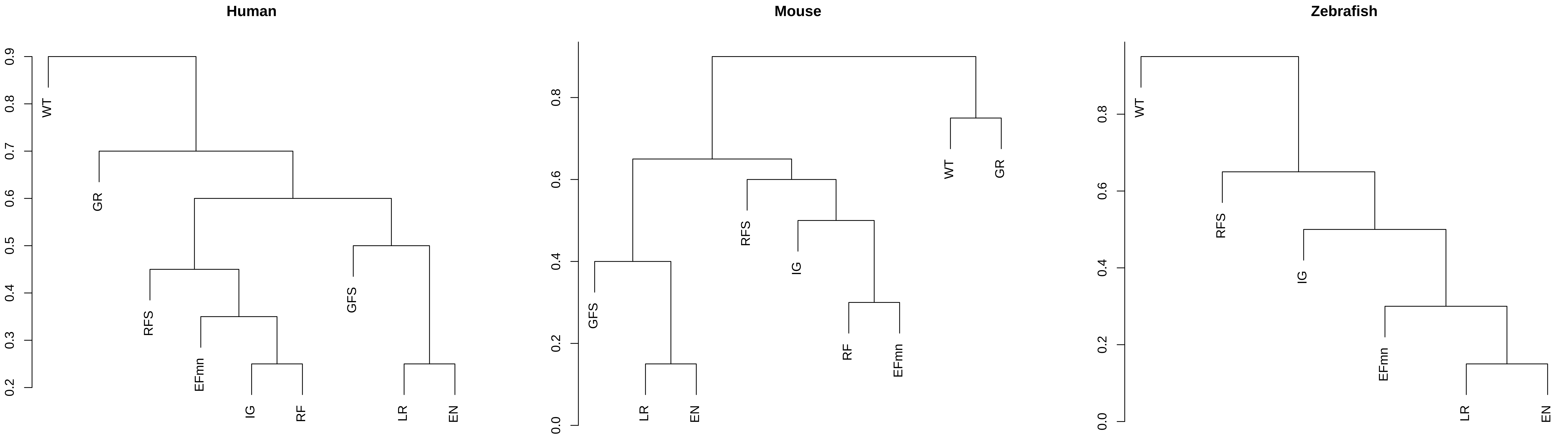

Figure S4. Hierarchical clusters of top 20 features selected by different algorithms computed with Jaccard distance and complete linkage
